# Epithelial cell lines of the cotton rat *(Sigmodon hispidus)* are highly susceptible *in vitro* models to zoonotic *Bunya*-, *Rhabdo*-, and *Flaviviruses*

**DOI:** 10.1186/s12985-016-0531-5

**Published:** 2016-05-04

**Authors:** Lukas Ehlen, Jan Tödtmann, Sabine Specht, René Kallies, Jan Papies, Marcel A. Müller, Sandra Junglen, Christian Drosten, Isabella Eckerle

**Affiliations:** Institute of Virology, University of Bonn Medical Centre, Sigmund-Freud-Strasse 25, 53127 Bonn, Germany; Institute for Medical Microbiology, Immunology & Parasitology (IMMIP), University of Bonn Medical Centre, Sigmund-Freud-Strasse 25, 53127 Bonn, Germany; Present address: Institute of Laboratory Animal Science, University of Zurich, Zurich, Switzerland; Present address: Helmholtz Centre for Environmental Research – UFZ, Leipzig, Germany

**Keywords:** *Sigmodon hispidus*, Cotton rat, Rodents, Cell culture model, Emerging viruses, Flaviviruses, Bunyaviruses, Rhabdoviruses, Zoonotic viruses

## Abstract

**Background:**

Small mammals such as bats and rodents have been increasingly recognized as reservoirs of novel potentially zoonotic pathogens. However, few in vitro model systems to date allow assessment of zoonotic viruses in a relevant host context. The cotton rat (*Sigmodon hispidus*) is a New World rodent species that has a long-standing history as an experimental animal model due to its unique susceptibility to human viruses. Furthermore, wild cotton rats are associated with a large variety of known or potentially zoonotic pathogens.

**Methods:**

A method for the isolation and culture of airway epithelial cell lines recently developed for bats was applied for the generation of rodent airway and renal epithelial cell lines from the cotton rat. Continuous cell lines were characterized for their epithelial properties as well as for their interferon competence. Susceptibility to members of zoonotic *Bunya*-, *Rhabdo-*, and *Flaviviridae,* in particular Rift Valley fever virus (RVFV), vesicular stomatitis virus (VSV), West Nile virus (WNV), and tick-borne encephalitis virus (TBEV) was tested. Furthermore, novel arthropod-derived viruses belonging to the families *Bunya*-, *Rhabdo-*, and *Mesoniviridae* were tested.

**Results:**

We successfully established airway and kidney epithelial cell lines from the cotton rat, and characterized their epithelial properties. Cells were shown to be interferon-competent. Viral infection assays showed high-titre viral replication of RVFV, VSV, WNV, and TBEV, as well as production of infectious virus particles. No viral replication was observed for novel arthropod-derived members of the *Bunya*-, *Rhabdo-*, and *Mesoniviridae* families in these cell lines.

**Conclusion:**

In the current study, we showed that newly established cell lines from the cotton rat can serve as host-specific in vitro models for viral infection experiments. These cell lines may also serve as novel tools for virus isolation, as well as for the investigation of virus-host interactions in a relevant host species.

## Background

Infectious diseases are a major threat to human health and remain among the leading causes of death and disability worldwide [1]. In the last decade, a variety of viruses such as Ebola virus, Hendra virus, Nipah virus, West Nile virus (WNV), and severe acute respiratory syndrome (SARS)- and Middle East respiratory syndrome (MERS)-coronaviruses have emerged or re-emerged, all of which are of zoonotic origin [2–5].

There have been a large number of novel, potentially zoonotic viruses that have been shown to be associated with small mammals, especially those of the orders *Chiroptera* and *Rodentia*, [4–14]. However, the isolation and propagation of these novel viruses has been unsuccessful in most instances, which limits further evaluation of their zoonotic risk.

Upon characterizing these novel viruses, it has become clear that most available animal models such as the domestic mouse or rat are of limited use, as they do not reflect the evolutionary conserved pathogen-host interaction that is a key trait of many reservoir-restricted viruses. In light of the large species range in which novel and potentially zoonotic viruses have been discovered, there remains a need for suitable in vitro models to understand virus-host interactions, interspecies spillover, and general viral pathogenicity [15]. Additionally, many of the natural reservoir hosts are protected or cannot be held in captivity, which limits in vivo studies in relevant hosts. Therefore, species-specific cell culture models may serve as acceptable surrogates [16–19].

The cotton rat (*Sigmodon hispidus*) is a unique example of a rodent species that is a well-established animal model to study viral pathogenesis and is also associated with a large range of zoonotic viruses in the wild [20–22]. Experimental studies in cotton rats have been performed for a large variety of human viruses, including important respiratory pathogens such as influenza or parainfluenza viruses, respiratory syncytial virus, and human metapneumovirus [23–33]. Furthermore, in the wild, cotton rats are associated with a variety of known or potential zoonotic viruses, such as classical rodent-borne viruses from the genera *Hantavirus* and *Arenavirus*, as well as members of the family *Flaviviridae*, such as WNV and St. Louis encephalitis virus (SLEV) [34–52].

To evaluate whether the broad viral susceptibility seen in both animalmodel and wild cotton rats was also reflected in *in vitro* cell culture models, we generated continuous cell lines from the respiratory and renal tracts of a cotton rat, and assessed their use for virus replication studies of known and potentially novel zoonotic viruses.

## Methods

### Generation of epithelial cell lines

Tissues from a laboratory-bred 3-month-old male cotton rat (*S. hispidus*) were kindly provided by the Institute for Medical Microbiology, Immunology and Parasitology (IMMIP), University of Bonn Medical Centre, Bonn, Germany. Ethical clearance was obtained from the respective authorities (No. AZ 84-02.05.20.13.114). The trachea and both kidneys of the euthanized cotton rat where removed *in toto*. All subsequent steps were then performed under sterile conditions using a laminar flow hood. Briefly, organ specimens were cleaned from surrounding tissue and then either sliced or roughly chopped with a sterile blade. Tissue slices were placed in 6-well cell culture plates at 37 °C in primary cell media. For tracheal cells, airway epithelial cell growth medium was used containing the following supplements: 0.004 mL/mL bovine pituitary extract, 10 ng/mL recombinant human epidermal growth factor, 5 μg/mL recombinant human insulin, 0.5 μg/mL hydrocortisone, 0.5 μg/mL epinephrine, 6.7 ng/mL triiodo-L-thyronine, 10 μg/mL human holo-transferrin, and 0.1 ng/mL retinoic acid (Promocell, Heidelberg, Germany). For kidney cells, renal epithelial cell growth medium was used containing the following supplements: 0.05 mL/mL foetal calf serum (FCS), 10 ng/mL recombinant human epidermal growth factor, 5 μg/mL recombinant human insulin, 36 ng/mL hydrocortisone, 0.5 μg/mL epinephrine, 4 pg/mL triiodo-L-thyronine, and 5 μg/mL human holo-transferrin (Promocell). Both media were supplemented with 1 % penicillin/streptomycin (Life Technologies GmbH, Darmstadt, Germany), 0.5 % of ofloxacin (Tarivid, Sanofi-Aventis, Frankfurt, Germany) and 1 % amphotericin B (PAA, Pasching, Austria) to avoid bacterial and fungal contamination during primary cell isolation and growth.

After the outgrowth of primary cells from organ specimens, the medium was changed every 2 days, and cell outgrowth was regularly observed. When nearly confluent, cells were immortalized by lentiviral transduction of the large T antigen of SV40 as described previously [17, 19, 53]. After immortalization, cells were passaged and stock-frozen until further use. All cell cultures were genotyped by polymerase chain reaction (PCR) amplification and sequencing of the mitochondrial cytochrome c oxidase subunit I and cytochrome b oxidase subunit I genes [54, 55]. To obtain single cell clones, cells were subcloned by end-point-limiting dilution and adapted to Dulbecco’s modified Eagle’s medium (DMEM) (PAA, Cölbe, Germany) with 4.5 g/L glucose (PAA), supplemented with 10 % FCS (PAA), 2 mM L-glutamine, 1 mM sodium pyruvate (PAA), nonessential amino acids (NEAA), 1 % penicillin/streptomycin (100X concentrate contains 10,000 units/mL penicillin and 10 mg/mL streptomycin) (Life Technologies), and 1 % amphotericin B as described previously [17, 19].

### Immunofluorescence assay

Cells were seeded on glass slides, and were washed the next day with PBS and fixed with acetone-methanol (1:1) for 5 min. Then, the acetone-methanol was removed and cells were washed again with PBS. Each slide was subsequently incubated overnight at 4 °C with 250 μL primary mouse monoclonal antibodies against pan-cytokeratin (Abcam ab7753, Cambridge, UK) and rabbit polyclonal antibodies against zonula occludens-1 (ZO-1 Mid) (Invitrogen 40–2200, Carlsbad, CA, USA) diluted 1:500 in PBS. Cells were washed and then incubated for 30 min at 25 °C with 125 μL cyanine 3 (Cy3)-labelled donkey-anti-mouse and Cy2-labelled donkey-anti-rabbit secondary antibodies (Dianova, Hamburg, Germany) diluted 1:500 in PBS. Cells were washed and then nuclei were counterstained with DAPI diluted at 1:1000 in PBS for 1 min. All images were obtained with a 207 Motic AxioVision microscope (Zeiss, Jena, Germany).

### Virus infection assays

Immortalized *S. hispidus* cells were seeded in 24-well plates at a density of 4 × 10^5^ cells/mL and grown in DMEM containing 5 % FCS and supplements as described above. The following day, cells were infected with vesicular stomatitis virus (VSV) strain Indiana or Rift Valley fever virus (RVFV) clone 13 at multiplicity of infections (MOIs) of 0.1 and 0.001 for both viruses. Cells were infected with WNV strain New York or tick-borne encephalitis virus (TBEV) strain K23 with MOIs of 0.01 and 0.001. Infectious units of the viral stocks and in the supernatant at the end of each experiment were determined by plaque-assays with Avicel overlays for RVFV and VSV as described previously [56], and with agarose overlays for WNV and TBEV as described previously [57].

For virus infection experiments, the medium was removed and cells were inoculated with virus diluted in Optipro serum-free medium (Life Technologies) for 1 h at 37 **°**C. Then, cells were washed twice with PBS. Growth medium was added and supernatants were harvested 0, 12 and 24 h after infection (hpi) for VSV; 0, 24 and 48 hpi for RVFV and 0, 6, 24 and 48 hpi after infection for WNV and TBEV. All virus infection experiments were performed in three individual replicates.

Viral RNA was extracted from cell culture supernatants with the Nucleospin RNA Virus kit according to the manufacturer’s instructions (Machery-Nagel, Düren, Germany). PCR was performed using the SuperScript III One-Step RT-PCR System with Platinum Taq DNA Polymerase (Invitrogen). Cycling conditions for VSV and RVFV quantitative reverse-transcription (qRT)-PCR were as follows: reverse transcription for 15 min at 55 °C, initial denaturation for 2 min at 95 °C, and 45 cycles of denaturation for 15 s at 95 °C and primer annealing/elongation for 30 s at 58 °C. Cycling conditions for WNV qRT-PCR were as follows: reverse transcription for 30 min at 45 °C, initial denaturation for 5 min at 95 °C, and 45 cycles of denaturation for 5 s at 95 °C and primer annealing/elongation for 35 s at 57 °C. qRT-PCR was carried out using the LightCycler 480 Real-Time PCR System (Roche, Basel, Switzerland). Primers and probes are available upon request.

To test the susceptibility of the *S. hispidus* cell lines to a variety of novel arthropod-derived viruses, cells were seeded in 24-well plates at a density of 4 × 10^5^ cells/mL. The following day, cells were infected with a titrated C6/36 cells-generated virus stock of Ferak [58], Moussa [59], or Cavally [60] virus at an MOI of 1.0. After infection, cells were observed daily for the presence of cytopathic effects (CPE). Supernatants from all infected cells were passaged onto fresh cells every 7 days for a total of three passages. Viral RNA was extracted from cell culture supernatants, and the presence of specific viral RNA was evaluated by qRT-PCR as described above.

### Assessment of interferon competence

To assess the interferon (IFN) competence of the cells, cells were seeded in 24-well plates at a density of 4 × 10^5^ cells/mL and grown in DMEM containing 10 % FCS and supplements as described above. The following day, cells were either transfected in triplicates with 1 μl of total RNA from VSV-infected cells (VSV-RNA) using the X-treme GENE siRNA transfection reagent (Roche, Basel, Switzerland) to stimulate the IFN response of the cells [61] or cells were left untreated as control. Eight hours after transfection, all cells were infected with the IFN-sensitive RVFV clone 13 carrying a *Renilla* luciferase [62]. 16 h after infection, cells were treated with lysis buffer and *Renilla* luciferase activity was measured in a microplate reader.

## Results

### *S. hispidus* and associated viruses

In order to assess the role of cotton rats as an experimental animal model for viral diseases and as a reservoir of zoonotic viruses in the wild, a review of the literature was performed. All studies that described cotton rats as experimental animal models for viral research, and all studies that described an association between viruses (via direct detection by PCR, or viral isolation in cell culture and antibody findings) and wild cotton rats were included (Table 1).

**Table 1:** Viruses associated with *S. hispidus* as experimental animal models or natural reservoir hosts (adapted and supplemented from that of Niewiesk et al. [22])

Virus family	Virus genus	Virus species	References
Experimental animal model			
*Adenoviridae*	*Mastadenovirus*	Human adenovirus C	[28, 30]
*Herpesviridae*	*Simplexvirus*	Herpes simplex virus type 1	[72]
*Orthomyxoviridae*	*Influenza virus A*	Avian and swine influenza viruses	[23, 24, 73]
	*Influenza virus B*		[24, 73]
*Paramyxoviridae*	*Metapneumovirus*	Human metapneumovirus	[25, 32, 33]
	*Morbillivirus*	Measles virus	[20, 74–77]
	*Pneumovirus*	Respiratory syncytial virus	[25, 29, 78]
	*Respirovirus*	Human parainfluenza virus type 3	[26, 27]
*Coronaviridae*	*Coronavirus*	Severe acute respiratory syndrome-associated coronavirus	[31]
*Picornaviridae*	*Enterovirus*	Poliovirus	[79, 80]
*Retroviridae*	*Lentivirus*	Human immunodeficiency virus type 1	[81]
Natural reservoir host			
*Arenaviridae*	*Arenavirus*	Guanarito virus	[47]
		Pirital virus	[38]
		Tamiami virus	[43–46]
		Whitewater Arroyo virus	[48]
*Bunyaviridae*	*Hantavirus*	Black Creek Canal virus	[34, 35]
		Bayou virus	[37]
		Muleshoe virus	[39]
	*Orthobunyavirus*	Zegla virus	[68]
		Jutiapa virus	[69]
*Flaviviridae*	*Flavivirus*	San Perlita virus	[69]
		St. Louis encephalitis virus	[51, 71]
		West Nile virus	[52]
		Cowbone Ridge virus	[70]
*Picornavirida*	*Cardiovirus*	Encephalomyocarditis virus	[50]
*Rhabdoviridae*	*Vesiculovirus*	Vesicular stomatitis virus	[49]
*Togaviridae*	*Alphavirus*	Highlands J virus	[51]
		Venezuelan equine encephalitis virus	[40–42]
		Eastern equine encephalitis virus	[36]

### Establishment of *S. hispidus* cell lines

**Fig. 1:**
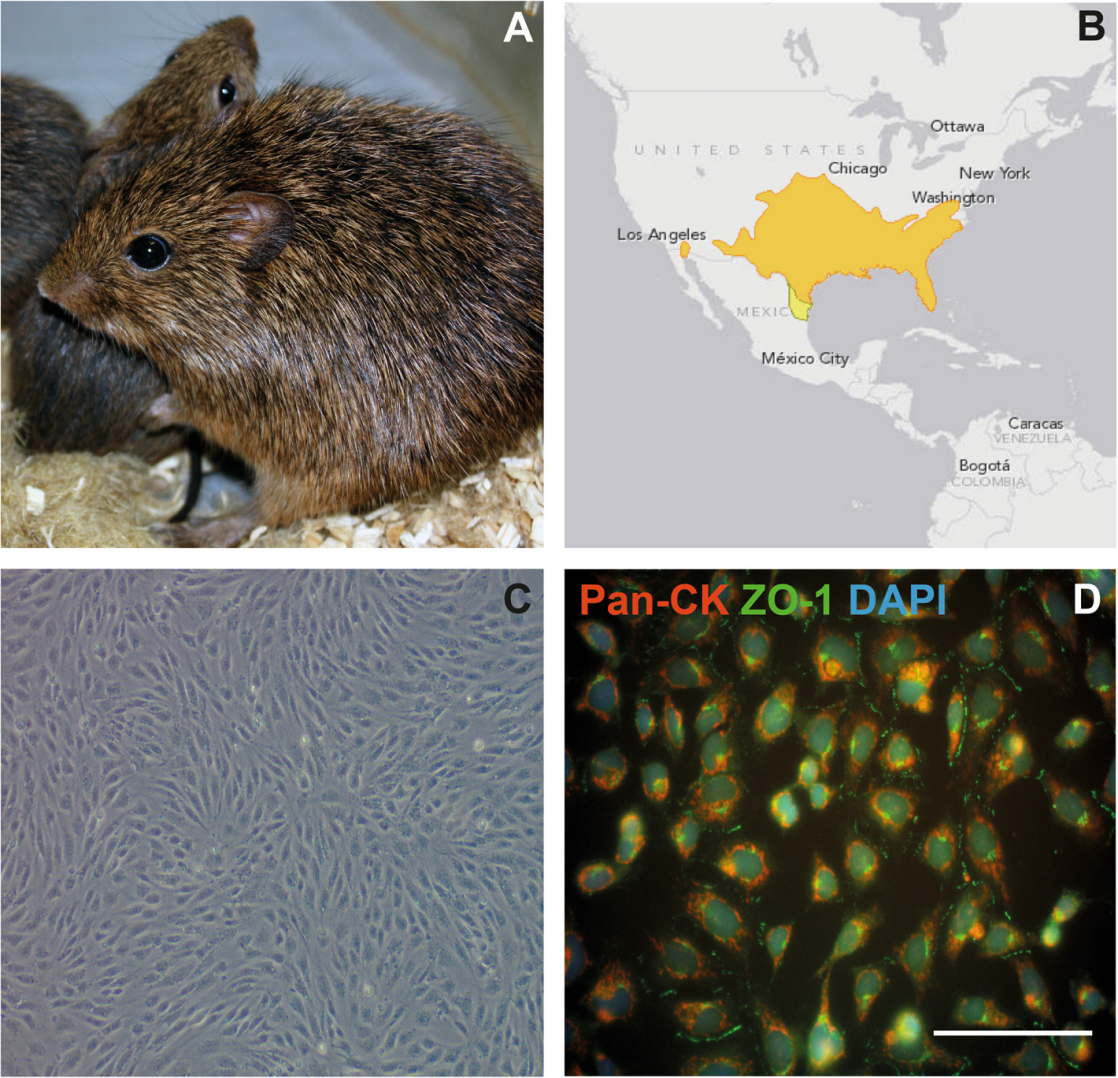
**a** Laboratory-bred cotton rats. **b** Distribution range of *S. hispidus* (map adapted from IUCN Red List of Threatened Species, http://www.iucnredlist.org). **c** Light microscopy image of subclone ShispREC.B-6. **d** Immunofluorescence staining for the following epithelial cell markers: pan-cytokeratin (Pan-CK) (red) and zonula occludens-1 (ZO-1) (green). Nuclei were counterstained with DAPI (blue)

Outgrowth of primary airway and kidney epithelial cells from cotton rat tissue samples was observed 3–5 days after the initiation of the cell culture. Outgrowing cells displayed a homogeneous, cobblestone-like morphology typical of epithelial cells in both the airway and renal epithelial cell cultures (Fig. 1). Successful immortalization was achieved by lentiviral transduction of the large T antigen of SV40 when the first patches of primary cells were visible in the cell culture dishes. Both airway epithelial (subsequently termed ShispAEC.B) and renal epithelial (subsequently termed ShispREC.B) cell lines showed rapid increases in cell growth 1–2 weeks after immortalization. To generate a homogeneous cell line, subclones were obtained and further characterized. By endpoint-limiting dilution, single-cell clones were selected and two subclones were used for further experiments, which were subsequently termed ShispAEC.B-2 and ShispREC.B-6. Both of these cell lines displayed epithelial cell morphology. The immortalized cell lines and the subclones generated in this study showed expression of pan-cytokeratin and zonula occludens 1 protein, confirming that the cells were of epithelial origin. Cytochrome b PCR amplification and sequencing of the product confirmed the host species (data not shown).

### Interferon competence of *S. hispidus* cell lines

**Fig. 2:**
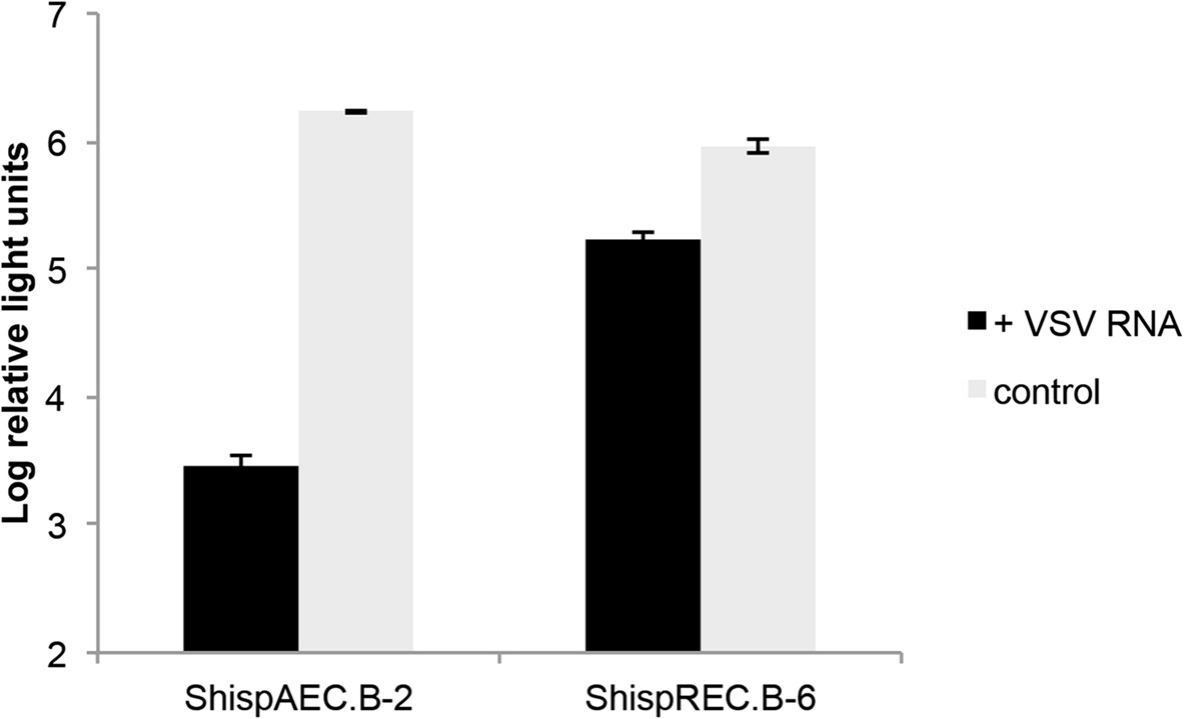
Interferon competence of ShispAEC.B-2 und ShispREC.B-6. Cells that were transfected with VSV-RNA for 8 h showed a reduced replication of RVFV 16 h after infection at an MOI of 0.02, as measured by production of Renilla luciferase in contrast to controls

ShispAEC.B-2 and ShispREC.B-6 were tested for their ability to respond to external stimulation of the interferon system. In order to stimulate the IFN response cells were transfected with total RNA from VSV-infected cells which was shown to trigger the RIG-I and MDA5-dependent IFN signalling cascade [61]. In comparison to untreated cells (Fig. 2, light column), VSV-RNA transfected cells (Fig. 2, dark columns) showed a 10-fold (renal cells) to 500-fold (airway cells) reduced replication of a highly IFN-sensitive RVFV-*Renilla* reporter virus. The pronounced decrease of RVFV-*Renilla* replication reflects the efficient induction of an antiviral state in both cell cultures. Overall, these data show that both subclones harbour an intact IFN response to external stimulation with airway cells showing a higher stimulation than renal epithelial cells.

### Infection of *S. hispidus* cells with VSV and RVFV

**Fig. 3:**
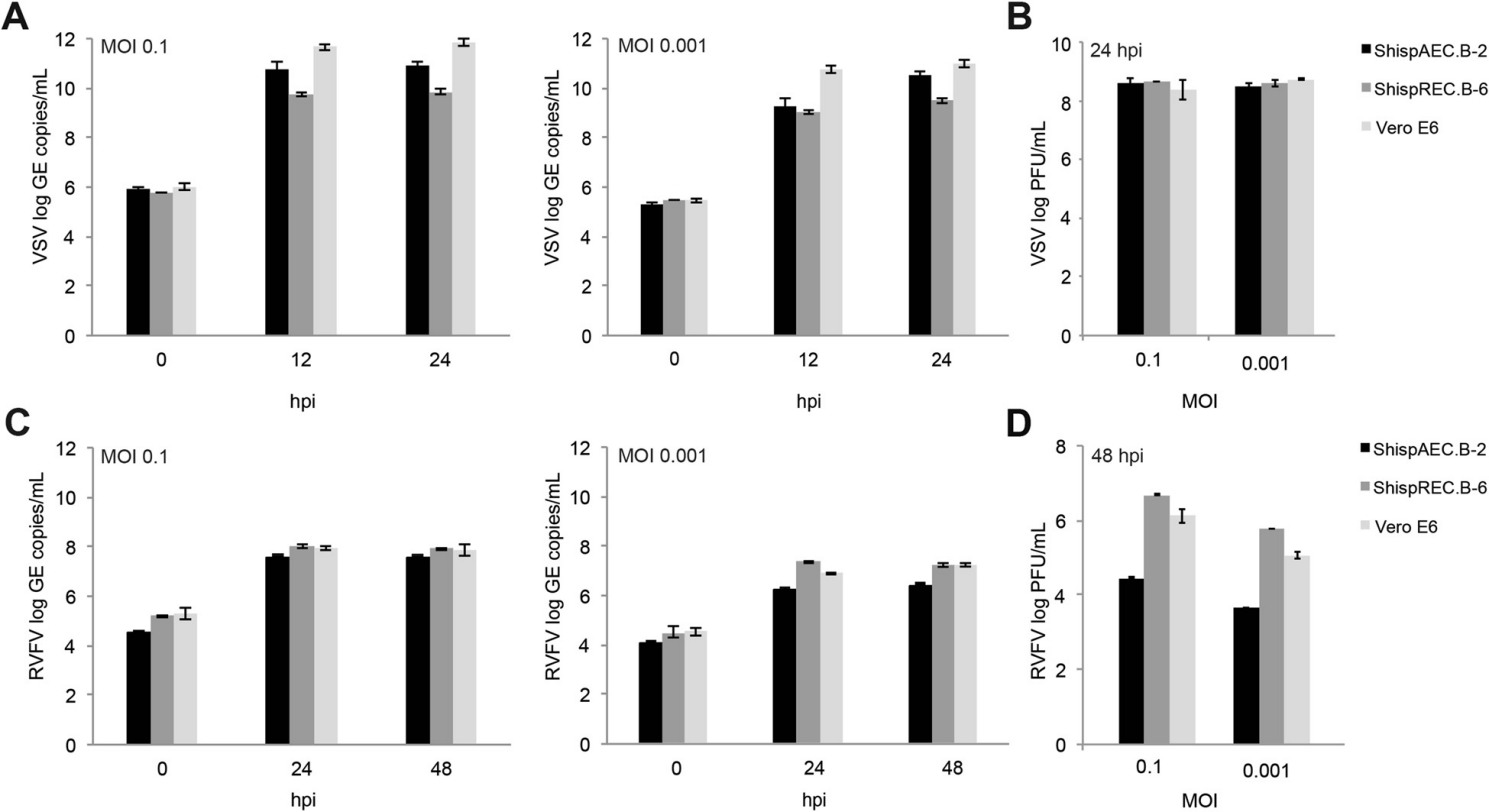
Viral infection studies with VSV (**a**, **b**) and RVFV (**c**, **d**) in subclones of immortalized *S. hispidus* airway epithelial cells (subclone 2, designated ShispAEC.B-2), *S. hispidus* renal epithelial cells (subclone 6, designated ShispREC.B-6), and Vero E6 cells. Viral replication was evaluated by qRT-PCR (**a**, **c**) and virus titration (**b**, **d**)

ShispAEC.B-2 and ShispREC.B-6 were infected with VSV and RVFV with two different MOIs, and the supernatants were harvested at different time points (Fig. 3). Vero E6 cells served as controls and were treated in parallel. Both cell lines exhibited a CPE and cell death after VSV and RVFV infection (data not shown). A 5.0 log increase in VSV viral RNA genome equivalent (GE) copies was seen after infection with an MOI of 0.1 for the airway epithelial cells, and a 4.1 log increase in GE copies was observed for the renal epithelial cells (Fig. 3a). Vero E6 cells showed an increase in GE copies of almost 6 log with the same experimental set-up. Upon infection with an MOI of 0.001, the maximum increases in log GE copies were approximately one log lower than those with an MOI of 0.1, with the highest GE copy numbers reached in Vero E6 cells (5.3 log increase), followed by the airway epithelial cells (4.1 log increase), and the renal epithelial cells (3.5 log increase). Production of infectious VSV particles was assessed 24 h after infection by titration of supernatants on Vero E6 cells, resulting in more than 8 log PFU/mL in all three cell lines after infection with a MOI of 0.1 and 0.001 (Fig. 3b).

Upon infection with RVFV at an MOI of 0.1, a maximum increase of 2.2 log in viral RNA GE copies was observed in the airway epithelial cells, and a 2.9 log increase in GE copies was seen for the renal epithelial cells. Vero E6 cells showed an increase of 2.7 log GE copies. Infections with a lower MOI of 0.001 showed comparable growth kinetics with slightly lower maximum increases in viral RNA (Fig. 3c). Production of infectious RVFV particles was assessed 48 h after infection by titration of supernatants on Vero E6 cells. The highest number of plaque-forming units was seen in ShispREC.B-6 with 6.7 log PFU/mL, followed by Vero E6 and ShispAEC.B-2 with 6.0 log and 4.5 PFU/mL after infection with an MOI of 0.1. Comparable results were observed after infection with a lower MOI resulting in 5.7; 5.1 and 3.7 log PFU/mL, respectively, for ShispREC.B-6, Vero E6 and ShispAEC.B-2 (Fig. 3d).

### Infection of *S. hispidus* cells with WNV and TBEV

**Fig. 4:**
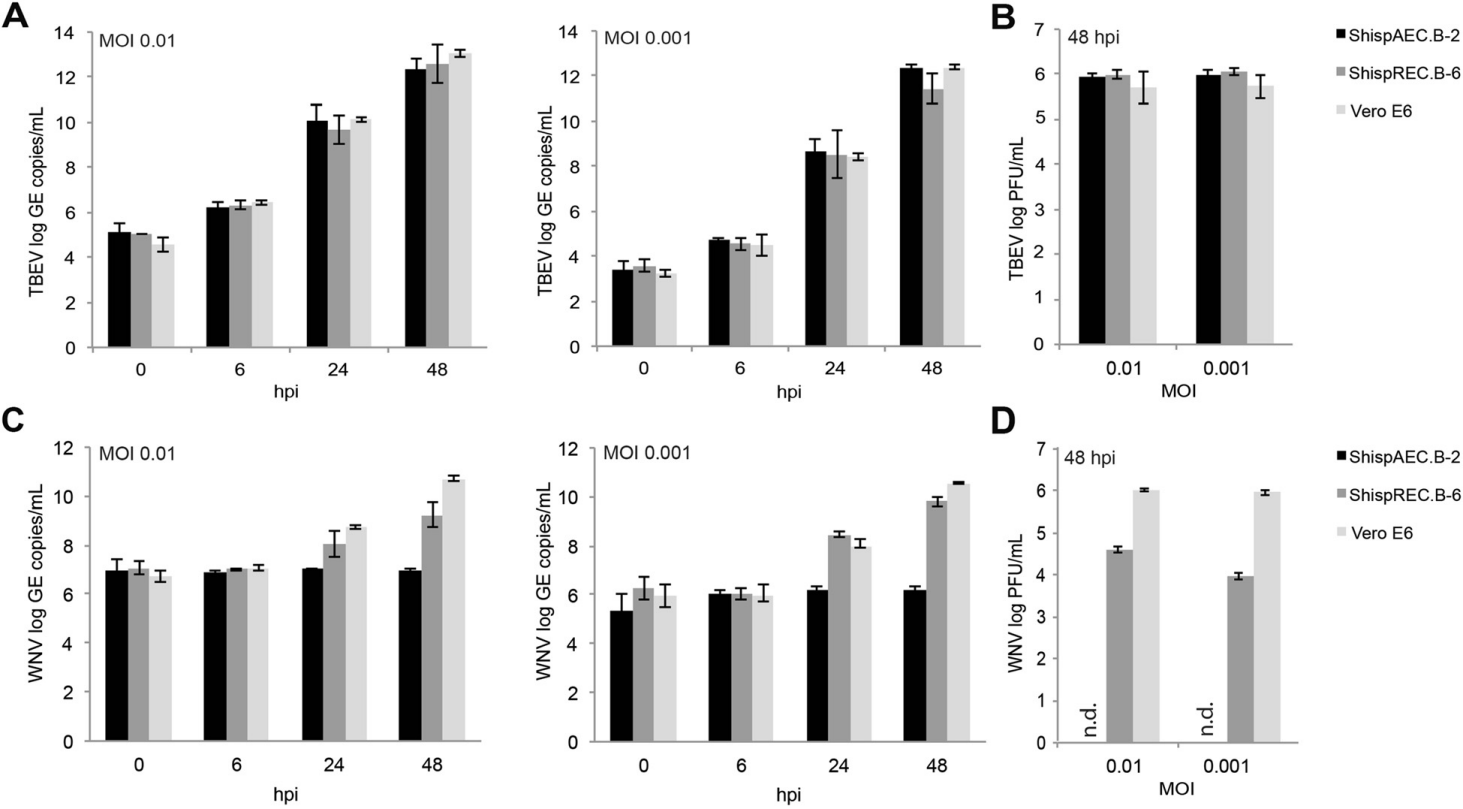
Viral infection studies with TBEV (**a**, **b**) and WNV (**c**, **d**) in subclones of immortalized *S. hispidus* airway epithelial cells (subclone 2, designated ShispAEC.B-2), *S. hispidus* renal epithelial cells (subclone 6, designated ShispREC.B-6), and Vero E6 cells. Viral replication was evaluated by qRT-PCR (**a**, **c**) and virus titration (**b**, **d**). n.d.; not detected

To assess *S. hispidus* cell susceptibility to viruses from the *Flaviviridae* family, infection experiments with WNV strain New York and TBEV were performed with two different MOIs, and the supernatants were collected at different time points. Vero E6 cells served as controls and were treated in parallel. Upon infection with TBEV, both *S. hispidus* cell lines and Vero E6 cells showed a CPE and rapid cell death within 48 h (data not shown). The maximum increase in viral RNA GE copies was 7.2 log for the airway epithelial cells, 7.6 log for the renal epithelial cells, and 8.4 log for Vero E6 cells after infection with a MOI of 0.01. Comparable growth kinetics were seen after infection with a MOI of 0.001. Production of infectious TBEV particles was assessed 48 h after infection by titration of supernatants on BHK-J cells. TBEV infectious particles were produced by all cell lines in comparable amounts of approximately 6 log PFU/mL after infection with a MOI of 0.01 and 0.001 (Fig. 4b).

For WNV, no increase in viral RNA was seen for the airway epithelial cells at either MOI. For the renal epithelial cells, a maximum increase of viral RNA GE copies of 2.2 log was observed, and a 4.0 log increase in GE copies was seen for Vero E6 cells. Comparable growth curves were seen for the lower MOI of 0.001 Production of infectious WNV particles was assessed 48 h after infection by titration of supernatants on BHK-J cells. The highest number of plaque-forming units was seen in VeroE6 cells with approximately 6 log PFU/mL, followed by lower titers in ShispREC.B-6 with 4.0 and 4.6 PFU/mL after infection with an MOI of 0.01 and 0.001, respectively. No production of infectious particles was seen in ShispAEC.B-2 cells (Fig. 4d).

### Infection of *S. hispidus* cells with novel insect-derived viruses

**Fig. 5:**
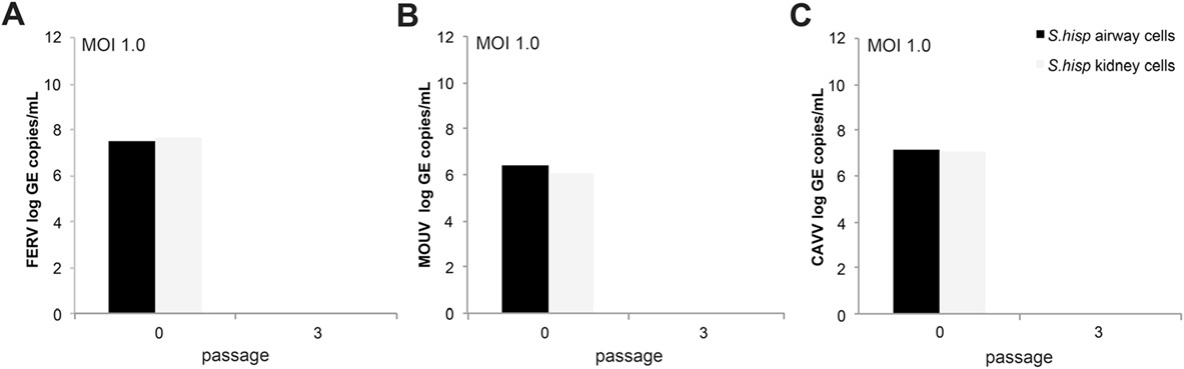
Susceptibilities of *S. hispidus* airway and kidney epithelial cells to novel arthropod-derived viruses were tested, including those of **a** Ferak virus (FERV), **b** Moussa virus (MOUV), **c** and Cavally virus (CAVV). Viral replication was determined by qRT-PCR

To assess the susceptibility of *S. hispidus* cell lines to members of the *Rhabdo-*, *Bunya-*, and *Mesoniviridae* families, further infection experiments were performed with recent novel virus isolates from insects [58–60]. Both airway and renal epithelial cells were inoculated with isolates of Moussa, Ferak, and Cavally viruses with a MOI of 1. No CPE was seen with daily observation. Supernatants were tested by viral specific qRT-PCR at the end of each passage, which did not reveal an increase in viral RNA, thus arguing against replication of these viruses in the cell lines generated in this study (Fig. 5).

## Discussion

In the work presented herein, we generated epithelial cell lines from the respiratory and renal tracts of a cotton rat due to its susceptibility to a broad range of human viruses, as well as the association of multiple important and emerging zoonotic viruses with this species.

*S. hispidus* is a rodent species with a long-standing history as an experimental animal model for virus research. Although the first animal experiments on cotton rats date back to the 1940s, only two cell lines from cotton rats are available to date. However, in contrast to experimental animals, cell lines are a less laborious model system, less expensive, and can be used in large-scale viral experiments such as in virus isolation trials without the ethical considerations that are involved in animal experiments. From the cotton rat, an osteoblastic cell line was previously derived from an osteogenic sarcoma (CCRT), of which two lymphoid cell lines (CR-T 1 and CR-T 2) were also derived [22]. However, these cell lines are used for the induction of tumours and as hybridoma cells to produce antibodies. No evaluation of these cells for their use in virus research has been performed despite a large range of viruses that have been investigated in *S. hispidus* animal experiments. Moreover, these cell lines are tumour cells that may not adequately resemble cells *in vivo* to study virus-host interactions, and they are not derived from target cells that are relevant to the natural course of a viral infection, such as epithelial cells. The value of species-specific cell lines has been shown particularity in the field of bat-borne viruses, where the use of bat cell lines has contributed significantly to studies of novel viruses, virus evolution, and virus adaptation during cell culture as well as replicative capacity and expression of host receptors [16, 53, 56, 63–65] (for a review see [18]). Cell lines derived from potential reservoir and intermediate hosts can serve as a valuable surrogate to study the replicative capacity of emerging zoonotic viruses, as has been demonstrated for the recently emerged viruses MERS-CoV and Ebola virus by work from our group [16, 56, 63, 66, 67].

In the current study, we evaluated the replicative capacity of viruses belonging to the four families *Bunyaviridae*, *Rhabdoviridae*, *Flaviviridae*, and *Mesoniviridae* in *S. hispidus* epithelial cell lines. Several members of the *Bunyaviridae* family were already shown to infect cotton rats, including Black Creek Canal virus (BCCV), which belongs to the genus *Hantavirus*. This virus was isolated from the lungs and spleens of cotton rats, and it was further shown by serologic analysis that *S. hispidus* was the primary rodent reservoir of BCCV [34, 35]. Other hantaviruses associated with *S. hispidus* are Bayou virus and Muleshoe virus [37, 39]. Additionally, from the genus *Orthobunyavirus*, an isolate termed Zegla virus was obtained from *S. hispidus* [68]. Here we showed that *S. hispidus* epithelial cells are highly susceptible to RVFV, a bunyavirus belonging to the genus *Phlebovirus*, with comparable growth kinetics to interferon-deficient Vero E6 cells. Furthermore, we tested the susceptibility of *S. hispidus* cells to a recently isolated bunyavirus termed Ferak virus that belongs to the sister taxon of the genus *Orthobunyavirus*. Interestingly, no growth of this virus was seen in the *S. hispidus* cell lines, suggesting an insect-specific replication cycle for this virus [58]. The further use of these cell lines for rodent-associated bunyaviruses such as hantaviruses should be evaluated in light of the promising findings for RVFV demonstrated herein.

For the *Rhabdoviridae* family, there have been serological findings in cotton rats that suggest a role for this species in the natural cycle of these viruses. Specifically, it was shown that neutralizing antibodies to both Indiana and New Jersey serotypes were found in *S. hispidus* in a VSV enzootic area in Costa Rica. Antibodies against either one or both serotypes were only found in *S. hispidus*, and not in exposed *Mus musculus* [49]. Our in vitro results showed that VSV replicates readily in *S. hispidus* cell lines with high replication titres of up to almost 11 log GE copies, which is approximately only one log lower than that of the replication titres seen in Vero E6 cells. These findings suggest that the *S. hispidus* cell culture models could serve as suitable in vitro models for further studies on VSV. To further assess the replication capacity of other rhabdoviruses in *S. hispidus* cells, the insect-derived Moussa virus was used [59]. Moussa virus was isolated from mosquitoes that also feed on mammals, but thus far, viral replication in human, hamster, or porcine cells has not been successful [59]. However, in line with the findings of a study by Quan et al., no replication of Moussa virus was seen in our experiments with *S. hispidus* cells. Additionally, another insect-derived isolate of a novel virus family termed *Mesoniviridae* was tested on our *S. hispidus* cell lines. Here, no replication of Cavally virus on the newly generated cells was seen.

A strong association has been reported between the cotton rat and several zoonotic flaviviruses, including WNV, SLEV, San Perlita virus, and Cowbone Ridge virus [51, 52, 69, 70]. Furthermore, cotton rats have been discussed as potential reservoir hosts in the wild for arboviruses, by which infected viremic cotton rats serve as a reservoir for arthropods that feed on them. In our cell culture experiments with TBEV and WNV, we saw replication and production of infectious virus particles in both *S. hispidus* cell lines for TBEV and in the kidney epithelial cells for WNV. Moreover, both replication titres were only one log lower than that for Vero E6 cells, indicating a high susceptibility of these cell lines to flaviviruses. Additionally, we have used *S. hispidus* cell lines for the evaluation of a novel sylvatic isolate of SLEV in an earlier study [71]. Here, it was shown that the endemic strain of SLEV, termed MSI-7, replicated in *S. hispidus* kidney cells. In contrast, the novel sylvatic SLEV isolate, termed Palenque strain, did not show any replication. As *S. hispidus* has been described as a natural reservoir host for SLEV, these findings suggest that the sylvatic isolate has not yet adapted to hosts that live outside the primary rain forest, whereas the endemic strain has [71]. In line with the findings obtained for SLEV, our results showed that WNV only replicated in kidney cells but not in airway epithelial cells, suggesting that kidney cells are more susceptible to this virus than airway cells. Taken together, the multiple in vitro findings presented herein for flaviviruses provide evidence that cotton rats may be reservoirs for multiple members of *Flaviviridae* in the wild*.* Therefore*, S. hispidus* cell lines, especially *S. hispidus* kidney epithelial cells, may provide a useful model for in vitro virus-host interaction studies.

## Conclusions

Newly generated epithelial cell lines from *S. hispidus* are able to support the replication of virus species from important zoonotic virus families, and may therefore serve as valuable tools for studies focusing on the isolation of novel viruses and virus-host interactions.
